# Effect of Glucagon-like Peptide-1 Receptor Agonists on Cardio-Metabolic Risk Factors among Obese/Overweight Individuals Treated with Antipsychotic Drug Classes: An Updated Systematic Review and Meta-Analysis of Randomized Controlled Trials

**DOI:** 10.3390/biomedicines11030669

**Published:** 2023-02-22

**Authors:** Dimitrios Patoulias, Theodoros Michailidis, Athina Dimosiari, Nikolaos Fragakis, Gary Tse, Manfredi Rizzo

**Affiliations:** 1 Second Department of Internal Medicine, European Interbalkan Medical Center, 57001 Thessaloniki, Greece; 2 Outpatient Department of Cardiometabolic Medicine, Second Department of Cardiology, Aristotle University of Thessaloniki, General Hospital “Hippokration”, 57001 Thessaloniki, Greece; 3 Second Department of Internal Medicine, Aristotle University of Thessaloniki, General Hospital “Hippokration”, 57001 Thessaloniki, Greece; 4 Kent and Medway Medical School, University of Kent and Canterbury Christ Church University, Kent CT2 7FS, UK; 5 Tianjin Key Laboratory of Ionic-Molecular Function of Cardiovascular Disease, Department of Cardiology, Tianjin Institute of Cardiology, Second Hospital of Tianjin Medical University, Tianjin 300211, China; 6 Department of Health Promotion, Mother and Child Care, Internal Medicine and Medical Specialties, School of Medicine, University of Palermo, 90133 Palermo, Italy

**Keywords:** GLP-1RA, psychosis, obesity, BMI, blood pressure, fasting glucose, lipid profile

## Abstract

Introduction: Glucagon-like peptide-1 receptor agonists (GLP-1RAs) constitute a drug class primarily developed for the treatment of subjects with type 2 diabetes, although they have also provided significant benefit for subjects with obesity without underlying diabetes. Individuals with psychotic disorders who are receiving antipsychotic treatment are a patient population at risk of developing obesity, which is linked to other metabolic disturbances. Methods: We searched PubMed and the Cochrane Library from inception to 1 December 2022, for randomized controlled trials (RCTs) enrolling obese or overweight adult subjects with an underlying psychotic disorder treated with antipsychotic drugs, randomized either to GLP-1RAs or a control. We set as the primary efficacy outcome the change in body weight and as secondary efficacy outcomes the change in body mass index (BMI) and in waist circumference, along with indices of glycemia, lipid profile, and blood pressure. Results: We pooled data from 4 trials (2 with liraglutide and 2 with exenatide) in a total of 199 enrolled subjects. GLP-1RA treatment, compared to control, resulted in a significant decrease in body weight by 3.8 kg [mean difference (MD) = −3.80, 95% CI; −6.35 to −1.24, I^2^ = 64%]. In addition, GLP-1RA treatment led to a significant decrease in BMI, compared to control, of 1.04 kg/m^2^ (MD = −1.04, 95% CI; −1.92 to −0.17, I^2^ = 35%). However, no significant effect on waist circumference was shown (MD = −3.2, 95% CI; −6.47 to 0.08, I^2^ = 88%). A significant improvement in glycemia and lipid profiles was also demonstrated with GLP-1RAs. No subgroup difference between liraglutide and exenatide was shown, and the use of GLP-1RAs did not increase the risk for treatment discontinuation compared to the control group. Conclusion: Treatment with GLP-1RAs can significantly improve weight loss and other cardiometabolic risk factors in obese people taking antipsychotic medications.

## 1. Introduction

It is well-established that individuals with psychotic disorders treated with antipsychotic drug classes feature an increased prevalence of overweight and obesity, with two-thirds of them being classified either as overweight or obese [1]. Antipsychotic drugs, except for ziprasidone, have been associated with a significant increase in body weight after a first episode of psychosis among the affected individuals [2]. Patients with underlying psychosis on the spectrum of schizophrenia experience a significantly increased risk for visceral and subcutaneous adiposity, which is associated with an impaired metabolic profile [3]. That adverse metabolic profile is associated with increased risk for type 2 diabetes mellitus (T2DM) [4], dyslipidemia [5], and cardiovascular disease (CVD) [6]. In addition, obesity among subjects with psychotic disorders has been associated with faster brain aging [7] and global cognitive impairment [8]. Therefore, prevention and management of obesity in that patient population are of great importance in order to prevent major complications and minimize morbidity and mortality.

It has been documented during the last decade that subjects with schizophrenia have shorter life expectancy compared to the general population; indeed, schizophrenia has been associated with a weighted average of 14.5 years of potential life lost, with an average life expectancy of 64.7 years [9]. According to a retrospective longitudinal cohort study from the United States with a sample of more than one million adult subjects with schizophrenia, this disease is associated with a significant increase in the risk for premature death compared to the general population, with CVD representing the leading cause of death among those individuals [10]. As a result, prompt identification of cardiovascular risk factors and aggressive treatment strategies are advised to reduce the mortality burden among those subjects [10]. Fortunately, treatment options that help control cardio-metabolic risk factors are now available in clinical practice.

Glucagon-like peptide-1 receptor agonists (GLP-1RAs) constitute a drug class primarily designed for the treatment of patients with T2DM, representing today’s cornerstone treatment approach for this disease. However, according to their safety and efficacy profiles, GLP-1RAs have also been utilized for the treatment of obesity, even in the absence of underlying T2DM [11,12,13]. Iqbal and colleagues recently demonstrated that GLP-1RAs compared to controls resulted in a significant decrease in body weight of 7.1 kg in a total of 11,459 enrolled subjects [12]. In another meta-analysis by Guo et al., in a total of 5867 enrolled obese subjects without concomitant T2DM, it was shown that GLP-1RAs compared to controls led to a significant weight loss of 5.39 kg [13]. 

Currently, liraglutide 3.0 mg once daily (Saxenda©) and semaglutide 2.4 mg once weekly (Wegovy©) from the class of GLP-1RAs have been officially approved by the Food and Drug Administration (FDA) for the therapeutic management of overweight and obesity. Liraglutide has been approved since December 2014 for weight management among obese individuals with a body mass index (BMI) greater than 30 kg/m^2^ or overweight subjects with a BMI greater than 27 kg/m^2^ with at least one related medical condition. On the other hand, semaglutide was approved for use in June 2021 as an additional drug to fight obesity. 

Of note, GLP-1RAs have also proved safe and efficacious in children and adolescents with obesity, leading to a mean reduction in body weight of 1.5 kg and in BMI of 1.24 kg/m^2^, along with improvements in the lipid profile and glycemic control, without major safety concerns [14]. 

Thus, this drug class might be an additional treatment option for subjects with obesity in the context of psychotic disorders and the use of antipsychotic drugs. As a result, we sought to assess the effect of GLP-1RAs on cardio-metabolic risk factors in overweight or obese individuals with psychotic disorders in this updated systematic review and meta-analysis, pooling data from relevant randomized controlled trials (RCTs). 

## 2. Methods

The present systematic review and meta-analysis were conducted according to the 2020 Preferred Reporting Items for Systematic Reviews and Meta-Analyses (PRISMA) guidelines [15]. The manuscript has been registered in the PROSPERO database (CRD42022382518).

***Databases***: we searched PubMed and Cochrane Library databases from their inception to December 1, 2022, in order to retrieve eligible RCTs assessing the safety and efficacy of GLP-1RAs in overweight or obese subjects with psychotic disorders treated with antipsychotic drug classes. 

***Inclusion criteria***: our inclusion criteria were: a. RCTs; b. enrollment of adult individuals; c. assignment to a GLP-1RA or standard of care (SOC); d. assessment of at least one cardio-metabolic parameter of interest. We excluded a. observational studies, b. case series, and c. potential trials enrolling adolescents. In addition, unpublished RCTs were excluded from our systematic review. 

***Search strategy***: we applied the following search strategy in both databases: (((((((((glucagon-like peptide-1 receptor agonist) OR (GLP-1 receptor analogue)) OR (GLP-1RA)) OR (liraglutide)) OR (exenatide)) OR (lixisenatide)) OR (dulaglutide)) OR (semaglutide)) OR (albiglutide)) OR (efpeglenatide) AND ((((psychosis) OR (antipsychotic drug)) OR (schizophrenia)) OR (schizoaffective disorder)) OR (bipolar disorder). We used only free-text words. We did not imply any filter regarding study setting, study sample, language, or publication date. 

***Outcomes of interest***: after de-duplication and assessment of potentially eligible studies for inclusion, two independent reviewers (D.P. and A.D.) extracted data of interest from the eligible reports. We set the change in body weight with GLP-1RAs versus SOC as the primary efficacy outcome. We set as secondary efficacy outcomes the corresponding changes in the following cardio-metabolic indices: BMI (in kg/m^2^), waist circumference (in cm), fasting plasma glucose (FPG, in mmol/L), office systolic blood pressure (SBP, in mm Hg), office diastolic blood pressure (DBP, in mm Hg), total cholesterol levels (TC, in mmol/L), low-density lipoprotein cholesterol levels (LDL-C, in mmol/L), high-density lipoprotein cholesterol levels (HDL-C, in mmol/L) and triglycerides levels (TRG, in mmol/L). Since the safety profile of GLP-1RAs is well-demarcated, we did not assess any safety outcomes in the present systematic review and meta-analysis, except for the risk of treatment discontinuation with GLP-1RAs versus control.

***Measurement of outcome***: concerning continuousvariables, we calculated for pre-specified outcomes of interest mean differences (MD), with a 95% confidence interval (CI), after implementation of the Mantel-Haenszel (M-H) random effects formula, while regarding dichotomousvariables, we calculated risk ratios (RR), with a 95% CI, after implementation of the M-H random effects formula. Statistical heterogeneity among studies was assessed using I^2^ statistics. All analyses were performed at the 0.05 significance level, and they were undertaken with RevMan 5.4.1 software.

***Risk of bias assessment***: two independent reviewers (D.P. and T.M.) assessed the quality of the included RCTs by using the Revised Cochrane risk of bias tool for randomized trials (RoB 2.0) for the primary efficacy outcome [16]. The RoB 2.0 tool assesses bias across the following domains: the randomization process, deviation from the intended intervention, missing outcome data, measurement of the outcome, and selection of the reported results. The risk-of-bias judgments for each domain are “low risk of bias,” “some concerns,” or “high risk of bias.” Discrepancies between reviewers were solved by discussion, consensus, or arbitration by a third senior reviewer (M.R.).

***Publication bias assessment***: we assessed publication bias by visual inspection of the corresponding funnel plot for the primary efficacy outcome (change in body weight). 

## 3. Results

**Figure 1:**
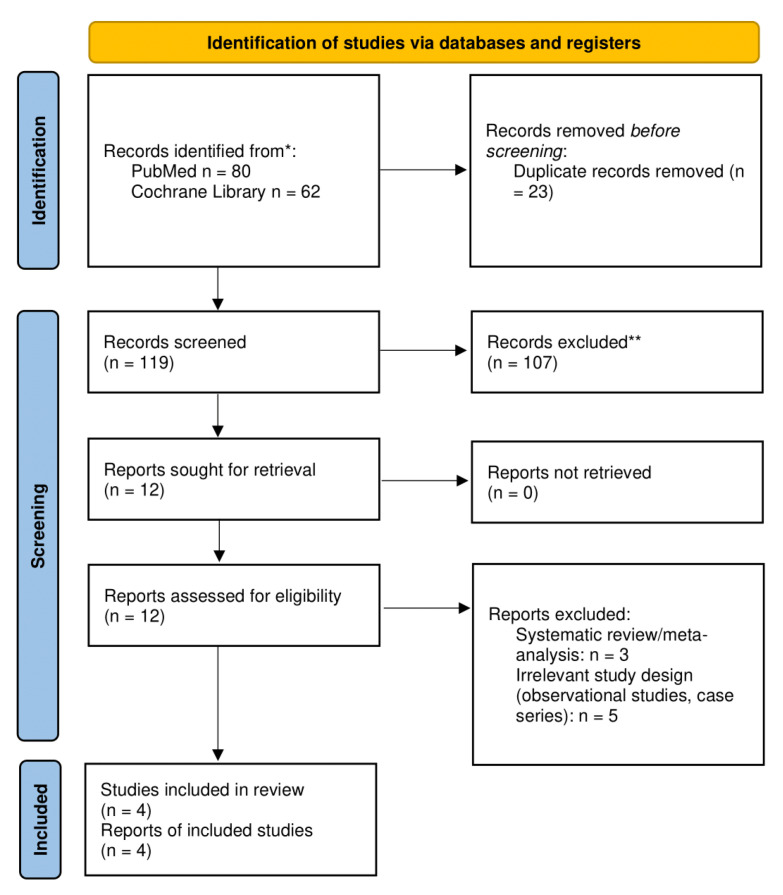
Flow diagram depicting the study selection process.

Out of 142 retrieved records, we assessed 12 of them for potential inclusion in our systematic review, finally reaching 4 eligible RCTs for inclusion in our quantitative synthesis [17,18,19,20]. The study selection process is depicted in Figure 1. Two trials utilized liraglutide versus SOC [17,19], while the remaining two trials compared exenatide with SOC [18,20]. Trials were conducted in Denmark, the United Kingdom, and Australia. Enrolled subjects were mainly diagnosed with schizophrenia or schizoaffective disorder. Mean BMI ranged from 33.7 to 39.5 kg/m^2^ in the GLP-1RA arms and from 33.9 to 41 kg/m^2^ in the control arms. Two trials [19,20] excluded subjects with co-morbid diabetes mellitus, while the relative numbers of subjects with underlying T2DM were relatively low in the two remaining trials [17,19]. A detailed summary of the main baseline characteristics of enrolled individuals across those RCTs is provided in Table 1. 

**Figure 2:**
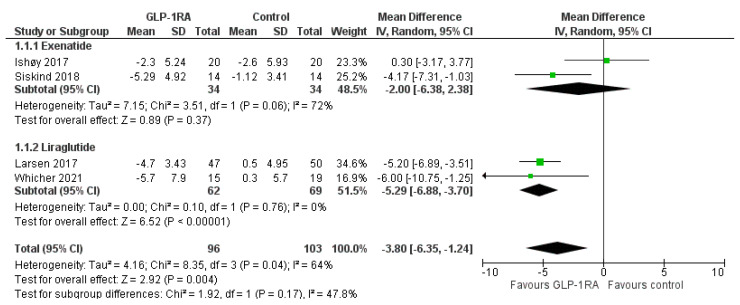
Effect of GLP-1RAs compared to controls on body weight among individuals treated with antipsychotic drugs. The diamond represents the overall effect estimate of the meta-analysis. The findings of each study are plotted as one square.

Regarding the primary efficacy outcome, we demonstrated that GLP-1RA treatment compared to control resulted in a significantly greater reduction in body weight by 3.8 kg (MD = −3.80, 95% CI; −6.35 to −1.24, I^2^ = 64%, *p* = 0.004), as shown in Figure 2. Sub-analysis according to the type of administered GLP-1RA showed that liraglutide compared to control decreased body weight by 5.29 kg (MD = −5.29, 95% CI; −6.88 to −3.70, I^2^ = 0%, *p* < 0.0001), while exenatide failed to produce a significant weight reduction (MD = −2.00, 95% CI; −6.38 to 2.38, I^2^ = 72%, *p* = 0.37). No significant subgroup difference was shown (p_subgroup_ = 0.17). 

**Figure 3:**
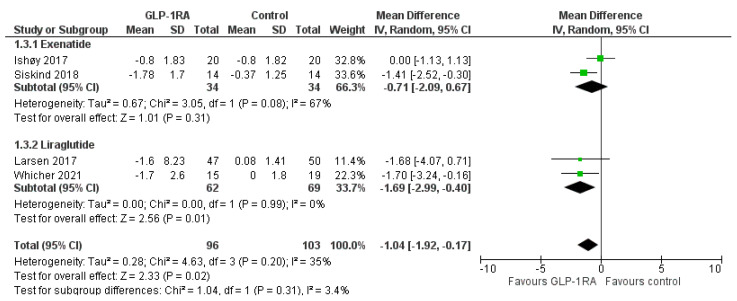
Effect of GLP-1RAs compared to controls on body mass index among individuals treated with antipsychotic drugs. The diamond represents the overall effect estimate of the meta-analysis. The findings of each study are plotted as one square.

**Figure 4:**
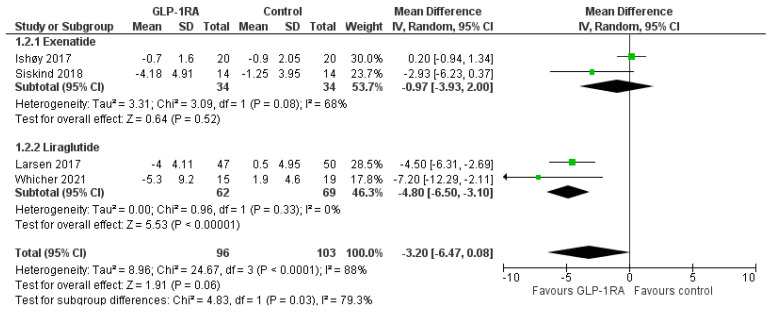
Effect of GLP-1RAs compared to controls on waist circumference among individuals treated with antipsychotic drugs. The diamond represents the overall effect estimate of the meta-analysis. The findings of each study are plotted as one square. he diamond represents the overall effect estimate of the meta-analysis. The findings of each study are plotted as one square.

In terms of BMI, GLP-1RA treatment compared to control led to a significant reduction of 1.04 kg/m^2^ (MD = −1.04, 95% CI; −1.92 to −0.17, I^2^ = 35%, *p* = 0.02), as shown in Figure 3. Again, liraglutide resulted in a significant reduction in BMI by 1.69 kg/m^2^ (MD = −1.69, 95% CI; −2.99 to −0.40, I^2^ = 0%, *p* = 0.01), whereas exenatide did not exert a significant effect on BMI compared to the control (MD = −0.71, 95% CI; −2.09 to 0.67, I^2^ = 67%, *p* = 0.31). No significant subgroup difference was shown (p_subgroup_ = 0.31). Treatment with GLP-1RA failed to produce a significant decrease in waist circumference compared to control (MD = −3.20, 95% CI; −6.47 to 0.08, I^2^ = 88%, *p* = 0.06), as demonstrated in Figure 4. Again, liraglutide resulted in a significant decrease in waist circumference by 4.8 cm (MD = −4.80, 95% CI; −6.50 to −3.10, I^2^ = 0%, *p* < 0.00001), while exenatide failed to produce a significant effect (MD = −0.97, 95% CI; −3.93 to 2.00, I^2^ = 68%, *p* = 0.52). A significant subgroup difference between the two treatment groups was documented (p_subgroup_ = 0.03).

**Figure 5:**
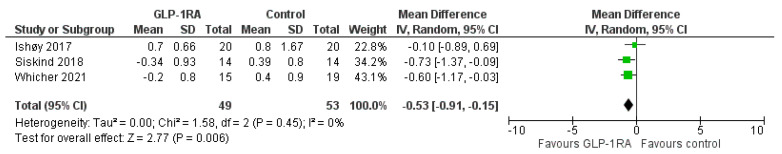
Effect of GLP-1RAs compared to control on fasting plasma glucose among individuals treated with antipsychotic drugs.

Concerning FPG, we pooled data from 3 RCTs that provided relevant data of interest. Overall, treatment with GLP-1RA compared to control resulted in a significant decrease in FPG by 0.53 mmol/L (MD = −0.53, 95% CI; −0.91 to −0.15, I^2^ = 0%, *p* = 0.006), as demonstrated in Figure 5. Similarly, concerning blood pressure levels, we also pooled data from 3 RCTs that assessed office blood pressure. More specifically, GLP-1RA treatment compared to control resulted in a non-significant effect on office SBP (MD = −1.44, 95% CI; −5.38 to 2.50, I^2^ = 0%, *p* = 0.47), as shown in Supplementary Figure S1. Similarly, GLP-1RA treatment compared to control failed to produce a significant decrease in office DBP (MD = −1.35, 95% CI; −5.62 to 2.91, I^2^ = 33%, *p* = 0.53), as depicted in Supplementary Figure S2.

Regarding lipid profile parameters, we documented that treatment with GLP-1RA compared to control led to a significant decrease in TC levels by 0.46 mmol/L (MD = −0.46, 95% CI; −0.70 to −0.21, I^2^ = 21%, *p* = 0.0002), as demonstrated in Supplementary Figure S3. In addition, treatment with GLP-1RA compared to control produced a significant increase in HDL-C levels by 0.09 mmol/L (MD = 0.09, 95% CI; 0.01 to 0.17, I^2^ = 0%, *p* = 0.03), as shown in Supplementary Figure S4, and a significant decrease in LDL-C levels by 0.31 mmol/L (MD = −0.31, 95% CI; −0.46 to −0.16, I^2^ = 0%, *p* < 0.0001), as shown in Supplementary Figure S5. Concerning TRG levels, GLP-1RA failed to provide a significant effect compared to control (MD = 0, 95% CI; −0.35 to 0.35, I^2^ = 0%, *p* = 1), as shown in Supplementary Figure S6.

Concerning the risk for treatment discontinuation due to adverse events, we did not observe any significant difference between GLP-1RAs and controls (RR = 1.26, 95% CI; 0.43 to 3.69, I^2^ = 34%, *p* = 0.68), as shown in Supplementary Figure S7.

Regarding the primary efficacy outcome, visual inspection of the corresponding funnel plot revealed the presence of asymmetry, possibly indicative of publication bias (Supplementary Figure S8), although, according to Cochrane recommendations, as a rule of thumb, tests for funnel plot asymmetry should be used only when there are at least 10 studies included in the meta-analysis.

Overall, the risk of bias across the selected RCTs included in the present systematic review with meta-analysis is considered low (Table 2).

## 4. Discussion

The present systematic review and meta-analysis of all relevant available RCTs documented that administration of GLP-1RAs among individuals with psychotic disorders treated with antipsychotic drugs is associated with A. a significant reduction in body weight and BMI, B. a significant reduction in FPG, and C. a significant improvement in lipid profile parameters (reduction in TC and LDL-C levels and increase in HDL-C levels). However, no effect of GLP-1RAs on waist circumference, office SBP, DBP, or TRG levels was shown. Of note, when the clinical efficacy of different GLP-1RAs was assessed, we demonstrated that liraglutide, but not exenatide, exhibited a favorable effect on cardio-metabolic parameters of interest in that patient population. However, no statistically significant subgroup difference between liraglutide and exenatide was shown (with the exception of waist circumference, for which the overall effect of GLP-1RAs versus control was non-significant).

From a physiologic perspective, it is well known that GLP-1 receptors (GLP-1R) are widely expressed in the brain. The GLP-1R rs1042044 gene polymorphism has been genetically linked to anhedonia but not to major depression diagnoses; unfortunately, no such data exist for psychotic disorders [21]. As shown in a post-mortem study, no abnormal gene expression of GLP-1R has been found in the brains of subjects with psychotic disorders, in contrast to the corresponding findings in patients with mood disorders [22]. In a previous analysis of data retrieved from 260 subjects with their first episode of psychosis, it was shown that GLP-1 and glucagon levels were significantly decreased in the whole sample of patients, representing the metabolism regulators mostly altered in the whole cohort [23]. Therefore, there might be an association between psychosis and impaired incretin secretion, whereas no association between GLP-1R expression and its polymorphisms has been related to the occurrence of psychotic disorders.

At a clinical level, overweight and obesity represent a major challenge in the management of subjects with psychotic disorders encountered in clinical practice; most cases are attributed to antipsychotic drug classes; however, it has also been demonstrated that never-treated or minimally treated subjects have a significantly elevated waist-to-hip ratio [24]. Interestingly, even in the first episode of psychosis, before initiation of antipsychotic drugs, affected subjects experience impaired appetite regulation in terms of significantly elevated insulin and decreased leptin levels, possibly correlating with negative emotional feelings [25]. Antipsychotic switching may improve the body weight profile and cardiometabolic health outcomes in some cases; however, the risk of psychiatric symptoms worsening should always be considered [26].

Lifestyle interventions to reduce body weight and improve cardiometabolic health have been shown to be effective in people with severe mental illness who are obese [27,28]. However, their efficacy at a numeric level seems to be rather small; lifestyle interventions with a duration less than 6 months resulted in a significant decrease in body weight by 0.2 kg, while those interventions applied for more than 12 months resulted in a significant decrease in body weight by 0.24 kg [28]. In addition, their implementation is not always easy in daily clinical practice, while consistency and maintenance of the achieved weight loss are challenging.

In terms of pharmacotherapy, a former meta-analysis documented that metformin, a drug that remains the first-line treatment option in T2DM, results in significant weight and BMI reduction in this population without affecting FPG [29]. Metformin leads to a significant weight loss of 3.24 kg compared to control and a significant BMI reduction of 1.11 kg/m^2^, also significantly reducing the insulin resistance index [29]. Other pharmacological approaches have also been implemented, with mild or no effect on weight reduction [30,31].

Evidence of anti-obesity effects of GLP-1RAs is solid and robust, especially with liraglutide and semaglutide [12,13]. Liraglutide 3.0 mg once daily and semaglutide 2.4 mg once weekly have been approved by the FDA for the treatment of obesity, even in the absence of T2DM as a co-morbidity, since 2014 and 2021, respectively. A previously published individual participant data meta-analysis of the first 3 published RCTs demonstrated that GLP-1RAs provided a significant weight reduction of 3.71 kg among subjects treated with antipsychotic drugs, with weight loss being greater for those patients treated with clozapine or olanzapine versus other antipsychotic drugs [32]. With the present meta-analysis, we update formerly published evidence, also highlighting the therapeutic utility of liraglutide but not of exenatide in subjects with underlying psychotic disorders and drug-induced obesity. Liraglutide appears to be an acceptable treatment option for obesity in this population, associated with significant improvements in cardio-metabolic risk factors and overall quality of life [33]. In addition, experimental data have been supportive of an antipsychotic effect of this agent [34], although relevant trials with liraglutide [17,19] failed to show any significant difference in psychiatric rating scales with liraglutide treatment versus control.

Currently, there is no evidence concerning the role of semaglutide on body weight control among subjects with psychotic disorders, except for a single case report [35]. However, based on their safety and efficacy profiles and their cardiovascular and renal efficacy [36,37], as well, it seems that GLP-1RAs, and especially liraglutide, semaglutide, and dulaglutide, should be preferred for weight management in this patient population. Regarding their safety, GLP-1RAs have been linked to mild, mostly transient gastrointestinal adverse events (nausea, vomiting, and diarrhea), while no major safety issues have arisen with their use [12,13]. Based on the well-demarcated side effects of GLP-1RAs, the present meta-analysis did not address safety outcomes in this specific population.

Overall, in the obesity population, GLP-1RAs appear as an efficacious and safe treatment option; however, in cases of morbid obesity, they seem to be inferior to bariatric surgery, which remains the treatment of choice [38]. However, since subjects with psychotic disorders have increased cardiovascular risk compared to healthy controls [6], while they also encounter substantial disparities in terms of CVD screening and treatment [39], it seems that GLP-1RAs appear as a reasonable treatment option, aiming at improving the cardio-metabolic health of those patients.

Regarding the comparison between GLP-1RAs and other treatment options with similar efficacy in terms of weight loss in this population, such as metformin [29], it seems that the use of GLP-1RAs should be prioritized due to their pleiotropic effects, including cardio- and reno-protective actions [36,37], if tolerated, and, of course, if they are affordable. In addition, once-weekly dosing regimens of some GLP-1RAs (such as exenatide, but also dulaglutide and semaglutide) might also be more efficacious as a treatment strategy, since they may enhance the compliance of patients to treatment, compared to once- or twice-daily administered pharmacologic interventions.

Our meta-analysis results in terms of body weight loss are in line with previous meta-analyses of obese subjects without diagnosed psychotic disorders [12,13]. Furthermore, our findings on lipid profile parameters are consistent with previous meta-analyses examining the impact of GLP-1RAs on lipid profiles in T2DM and non-alcoholic fatty liver disease [40,41]. Finally, we failed to document a significant effect of GLP-1RAs on office SBP and DBP among obese subjects with psychotic disorders, a finding similar to a previous meta-analysis performed by some of us in the T2DM population [42]. As a result, regardless of T2DM status at baseline, the effect of GLP-1RAs on cardio-metabolic risk factors appears to be similar in subjects with and without concomitant psychotic disorder.

We consider the main limitations of the present meta-analysis to be the small number of included RCTs and the limited number of enrolled subjects, along with the absence of individual participant data in order to conduct further subgroup analyses according to other baseline characteristics of interest. Furthermore, none of the eligible RCTs used semaglutide, a GLP-1RA that has been shown to significantly increase the odds of significant weight loss >5% compared to baseline and body weight change [43,44]. Results of two relevant RCTs, registered at clinicaltrials.gov (NCT04892199 and NCT05333003), are awaited in order to determine whether semaglutide can also be useful for the treatment of obesity among individuals with an underlying psychotic disorder. Of note, lack of access to individual participants’ data did not permit sensitivity analyses according to baseline characteristics of interest. Finally, the presence of publication bias is suspected based on visual inspection of the funnel plot for the primary efficacy outcome; however, the limited number of eligible RCTs does not ensure an accurate assessment of publication bias, as stated previously, according to Cochrane recommendations.

## 5. Conclusions

GLP-1RAs appear to be an efficacious treatment option for weight management in individuals with obesity related to antipsychotic drugs, also improving glycemia and lipid profile parameters. Future trials with semaglutide are awaited in order to provide definitive answers for this interesting and challenging treatment issue.

## Figures and Tables

**Table 1:** Participants’ baseline characteristics across the eligible trials.

	Ishøy et al.	Siskind et al.	Larsen et al.	Whicher et al.
Country	Denmark	Australia	Denmark	United Kingdom
Number of randomized subjects	45	28	103	47
Number of analyzed subjects after dropouts	40	28	97	34
Study design	Double-blind	Open label	Double-blind	Double-blind
Assigned glucagon-like peptide-1 receptor agonist	Exenatide 2 mg once weekly	Exenatide 2 mg once weekly	Liraglutide up to 1.8 mg once daily	Liraglutide up to 3.0 mg once daily
Treatment duration, in weeks	12	24	16	24
Diagnosed psychotic disorder	**Schizophrenia**: 36 (90%) **Schizoaffective disorder**: 4 (10%)	**Schizophrenia**: 28 (100%)	**Schizophrenia**: 96 (98.9%) **Psychosis**: 1 (1.1%)	**Schizophrenia**: 28 (59.6%) **Schizoaffective disorder**: 17 (36.2%) **First episode of psychosis**: 2 (4.2%)
Age, in years	**Exenatide**: 37.4 (10.7) **Control**: 34.4 (10.6)	**Not reported**	**Liraglutide**: 42.1 (10.7) **Control**: 43.0 (10.5)	**Liraglutide**: 42.7 (11.3) **Control**: 45.4 (10.7)
Sex, male/female	**Exenatide**: 11/9 **Control**: 9/11	**Exenatide**: 11/3 **Control**: 7/7	**Liraglutide**: 30/17 **Control**: 30/20	**Liraglutide**: 15/9 **Control**: 9/14
Body mass index, in kg/m^2^	**Exenatide**: 39.5 (3.5) **Control**: 38.6 (6.3)	**Exenatide**: 35.56 (2.42) **Control**: 35.80 (3.77)	**Liraglutide**: 33.7 (5.1) **Control**: 33.9 (6.6)	**Liraglutide**: 37.5 (6.9) **Control**: 41.0 (6.7)
Baseline diabetes mellitus, *n* (%)	Exclusion criterion	**Exenatide**: 3 (21.4%) **Control**: 2 (14.3%)	Exclusion criterion	**Liraglutide**: 1 (4%) **Control**: 3 (14%)
Registration number	EudraCT no. 2012-005404-17	ACTRN12615000524594	NCT01845259	EudraCT no. 2017-004064-35

**Table 2:** Assessment of the risk of bias across the selected trials.

Study	Randomization Process	Deviations from Intended Interventions	Missing Outcome Data	Measurement of the Outcome	Selection of the Reported Result	Overall
Ishøy et al.	Low	Low	Low	Low	Low	Low
Siskind et al.	Low	Low	Low	Low	Unclear	Low
Larsen et al.	Low	Low	Low	Low	Low	Low
Whicher et al.	Low	Low	Low	Low	Low	Low

## Data Availability

Data available upon reasonable request from authors.

## Supplementary Materials

The following supporting information can be downloaded at: https://www.mdpi.com/article/10.3390/biomedicines11030669/s1, Figure S1: Effect of GLP-1RAs compared to control on office systolic blood pressure among individuals treated with antipsychotic drugs; Figure S2: Effect of GLP-1RAs compared to control on office diastolic blood pressure among individuals treated with antipsychotic drugs; Figure S3: Effect of GLP-1RAs compared to control on total cholesterol levels among individuals treated with antipsychotic drugs; Figure S4: Effect of GLP-1RAs compared to control on LDL-cholesterol levels among individuals treated with antipsychotic drugs; Figure S5: Effect of GLP-1RAs compared to control on HDL-cholesterol levels among individuals treated with antipsychotic drugs; Figure S6: Effect of GLP-1RAs compared to control on triglycerides levels among individuals treated with antipsychotic drugs; Figure S7: Risk for treatment discontinutation due to adverse events with GLP-1RAs compared to control among individuals treated with antipsychotic drugs; Figure S8: Funnel plot for visual inspection of publication bias.
