# Supplementary material for: Effect of Glucagon-like Peptide-1 Receptor Agonists on Cardio-Metabolic Risk Factors among Obese/Overweight Individuals Treated with Antipsychotic Drug Classes: An Updated Systematic Review and Meta-Analysis of Randomized Controlled Trials
Source: Biomedicines. 2023 Feb 22;11(3):669. doi: 10.3390/biomedicines11030669 (PMC10045529; doi:10.3390/biomedicines11030669)
Supplement: Supplementary file 1 [file biomedicines-11-00669-s001.zip › biomedicines-2140946-supplementary.pdf]

Study or Subgroup	GLP-1RA Mean	GLP-1RA SD	GLP-1RA Total	Control Mean	Control SD	Control Total	Weight	Mean Difference IV, Random, 95% CI
Larsen 2017	-1.4	13.71	47	1.1	12.73	50	55.9%	-2.50 [-7.77, 2.77]
Siskind 2018	-2.29	10.83	14	-0.71	8.54	14	29.8%	-1.58 [-8.80, 5.64]
Whicher 2021	-3	14	15	-6	17	19	14.3%	3.00 [-7.42, 13.42]
**Total (95% CI)**			**76**			**83**	**100.0%**	**-1.44 [-5.38, 2.50]**

Heterogeneity: Tau^2 = 0.00; Chi^2 = 0.85, df = 2 (P = 0.65); I^2 = 0%
Test for overall effect: Z = 0.72 (P = 0.47)

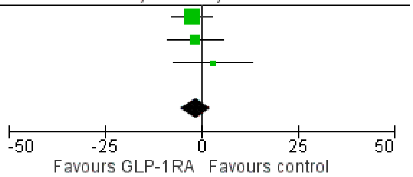

Figure S1. Effect of GLP-1RAs compared to control on office systolic blood pressure among individuals treated with antipsychotic drugs. The diamond represents the overall effect estimate of the meta-analysis. The findings of each study are plotted as one square.

Study or Subgroup	GLP-1RA Mean	GLP-1RA SD	GLP-1RA Total	Control Mean	Control SD	Control Total	Weight	Mean Difference IV, Random, 95% CI
Larsen 2017	0.5	10.28	47	2.4	7.78	50	55.4%	-1.90 [-5.55, 1.75]
Siskind 2018	-1.5	9.77	14	4.07	11.72	14	21.8%	-5.57 [-13.56, 2.42]
Whicher 2021	2	14	15	-2	7	19	22.8%	4.00 [-3.75, 11.75]
**Total (95% CI)**			**76**			**83**	**100.0%**	**-1.35 [-5.62, 2.91]**

Heterogeneity: Tau^2 = 5.08; Chi^2 = 2.98, df = 2 (P = 0.23); I^2 = 33%
Test for overall effect: Z = 0.62 (P = 0.53)

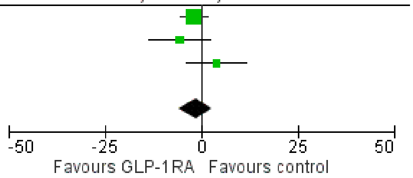

Figure S2. Effect of GLP-1RAs compared to control on office diastolic blood pressure among individuals treated with antipsychotic drugs. The diamond represents the overall effect estimate of the meta-analysis. The findings of each study are plotted as one square.

Study or Subgroup	GLP-1RA Mean	GLP-1RA SD	GLP-1RA Total	Control Mean	Control SD	Control Total	Weight	Mean Difference IV, Random, 95% CI
Ishøy 2017	0.7	1.48	20	0.8	1.14	20	8.3%	-0.10 [-0.92, 0.72]
Larsen 2017	-0.5	0.62	47	0.09	0.57	50	59.8%	-0.59 [-0.83, -0.35]
Whicher 2021	-0.3	0.6	15	0	0.5	19	31.9%	-0.30 [-0.68, 0.08]
**Total (95% CI)**			**82**			**89**	**100.0%**	**-0.46 [-0.70, -0.21]**

Heterogeneity: Tau^2 = 0.01; Chi^2 = 2.52, df = 2 (P = 0.28); I^2 = 21%
Test for overall effect: Z = 3.68 (P = 0.0002)

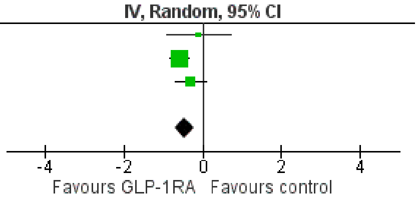

Figure S3. Effect of GLP-1RAs compared to control on total cholesterol levels among individuals treated with antipsychotic drugs. The diamond represents the overall effect estimate of the meta-analysis. The findings of each study are plotted as one square.

Study or Subgroup	GLP-1RA Mean	GLP-1RA SD	GLP-1RA Total	Control Mean	Control SD	Control Total	Weight	Mean Difference IV, Random, 95% CI
Ishøy 2017	0.2	0.27	20	0.1	0.23	20	27.2%	0.10 [-0.06, 0.26]
Siskind 2018	0.06	0.19	14	-0.01	0.17	14	36.9%	0.07 [-0.06, 0.20]
Whicher 2021	0	0.2	15	-0.1	0.2	19	35.9%	0.10 [-0.04, 0.24]
**Total (95% CI)**			**49**			**53**	**100.0%**	**0.09 [0.01, 0.17]**

Heterogeneity: Tau^2 = 0.00; Chi^2 = 0.12, df = 2 (P = 0.94); I^2 = 0%
Test for overall effect: Z = 2.15 (P = 0.03)

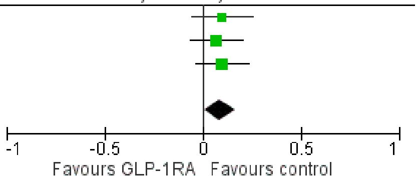

Figure S4. Effect of GLP-1RAs compared to control on LDL-cholesterol levels among individuals treated with antipsychotic drugs. The diamond represents the overall effect estimate of the meta-analysis. The findings of each study are plotted as one square.

Study or Subgroup	GLP-1RA Mean	GLP-1RA SD	GLP-1RA Total	Control Mean	Control SD	Control Total	Weight	Mean Difference IV, Random, 95% CI
Ishøy 2017	0.7	1.28	20	0.7	0.87	20	5.0%	0.00 [-0.68, 0.68]
Larsen 2017	-0.4	0.55	47	-0.06	0.35	50	67.1%	-0.34 [-0.52, -0.16]
Whicher 2021	-0.2	0.5	15	0.1	0.3	19	27.9%	-0.30 [-0.59, -0.01]
**Total (95% CI)**			**82**			**89**	**100.0%**	**-0.31 [-0.46, -0.16]**
Heterogeneity: Tau² = 0.00; Chi² = 0.91, df = 2 (P = 0.64); I² = 0%								
Test for overall effect: Z = 4.04 (P < 0.0001)								

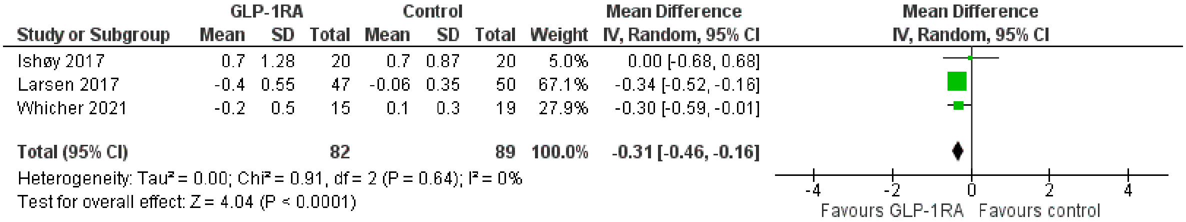

Figure S5. Effect of GLP-1RAs compared to control on HDL-cholesterol levels among individuals treated with antipsychotic drugs. The diamond represents the overall effect estimate of the meta-analysis. The findings of each study are plotted as one square.

Study or Subgroup	GLP-1RA Mean	GLP-1RA SD	GLP-1RA Total	Control Mean	Control SD	Control Total	Weight	Mean Difference IV, Random, 95% CI
Ishøy 2017	0.9	1.21	20	0.7	1.37	20	19.4%	0.20 [-0.60, 1.00]
Siskind 2018	-0.24	1.31	14	-0.01	0.95	14	17.3%	-0.23 [-1.08, 0.62]
Whicher 2021	0	0.8	15	0	0.4	19	63.3%	0.00 [-0.44, 0.44]
**Total (95% CI)**			**49**			**53**	**100.0%**	**-0.00 [-0.35, 0.35]**
Heterogeneity: Tau² = 0.00; Chi² = 0.52, df = 2 (P = 0.77); I² = 0%								
Test for overall effect: Z = 0.01 (P = 1.00)								

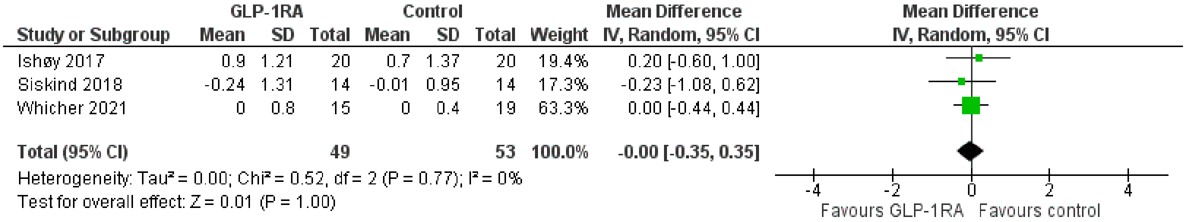

Figure S6. Effect of GLP-1RAs compared to control on triglycerides levels among individuals treated with antipsychotic drugs. The diamond represents the overall effect estimate of the meta-analysis. The findings of each study are plotted as one square.

Study or Subgroup	GLP-1RA Events	GLP-1RA Total	Control Events	Control Total	Weight	Risk Ratio M-H, Random, 95% CI
**1.11.1 Exenatide**						
Ishøy 2017	3	23	2	22	28.2%	1.43 [0.26, 7.78]
Siskind 2018	0	14	0	14		Not estimable
**Subtotal (95% CI)**		**37**		**36**	**28.2%**	**1.43 [0.26, 7.78]**
Total events	3		2			
Heterogeneity: Not applicable						
Test for overall effect: Z = 0.42 (P = 0.68)						
**1.11.2 Liraglutide**						
Larsen 2017	5	52	1	51	20.3%	4.90 [0.59, 40.53]
Whicher 2021	5	24	7	23	51.6%	0.68 [0.25, 1.85]
**Subtotal (95% CI)**		**76**		**74**	**71.8%**	**1.48 [0.21, 10.45]**
Total events	10		8			
Heterogeneity: Tau² = 1.38; Chi² = 2.94, df = 1 (P = 0.09); I² = 66%						
Test for overall effect: Z = 0.39 (P = 0.69)						
**Total (95% CI)**		**113**		**110**	**100.0%**	**1.26 [0.43, 3.69]**
Total events	13		10			
Heterogeneity: Tau² = 0.33; Chi² = 3.05, df = 2 (P = 0.22); I² = 34%						
Test for overall effect: Z = 0.42 (P = 0.68)						
Test for subgroup differences: Chi² = 0.00, df = 1 (P = 0.98), I² = 0%						

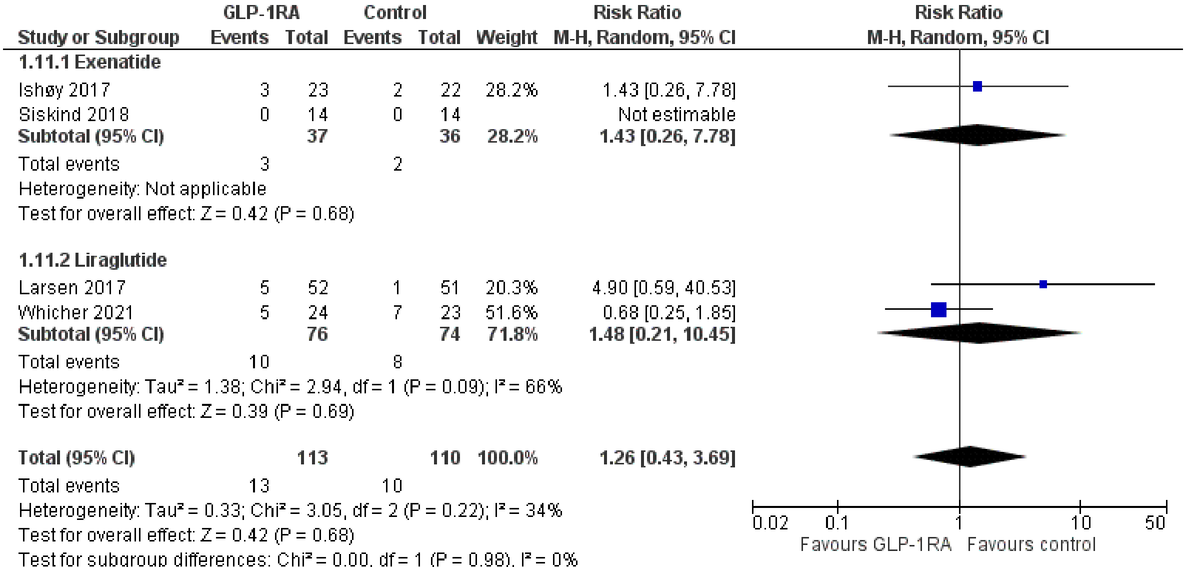

Figure S7. Risk for treatment discontinutation due to adverse events with GLP-1RAs compared to control among individuals treated with antipsychotic drugs. The diamond represents the overall effect estimate of the meta-analysis. The findings of each study are plotted as one square.

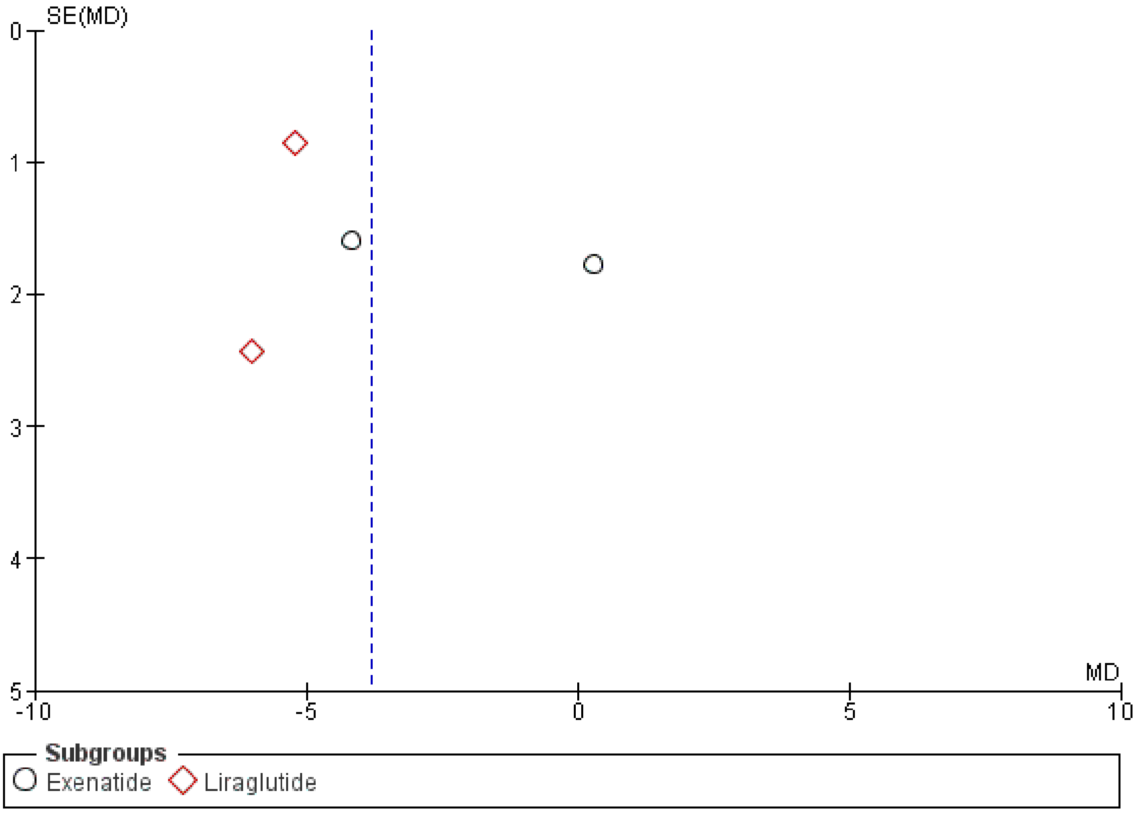

Figure S8. Funnel plot for visual inspection of publication bias.
